# Light People: Prof. Lei Zhou spoke about metasurfaces

**DOI:** 10.1038/s41377-025-01893-z

**Published:** 2025-07-02

**Authors:** Chenzi Guo

**Affiliations:** Changchun Institute of Optics, Fine Mechanics and Physics, Chinese Academy of Sciences, Changchun, China

**Keywords:** Nanophotonics and plasmonics, Metamaterials

## Abstract

Prof. Lei Zhou is a leading figure in metamaterials and metasurfaces. His pioneering works on developing gradient-index metasurfaces and utilizing ultrathin anisotropic materials for polarization control have co-shaped the foundational framework of metasurfaces. In addition to his research achievements, Prof. Zhou serves as Vice President of Fudan University - one of China’s most prominent universities - and was recently appointed as the Head of Fudan’s College of Future Information Technology. With such roles, he’s been deeply involved in many strategic initiatives and policies that contribute to the well-being of the scientific community. To shed light on the above, *Light: Science & Applications* invited Prof. Lei Zhou for an in-depth conversation.

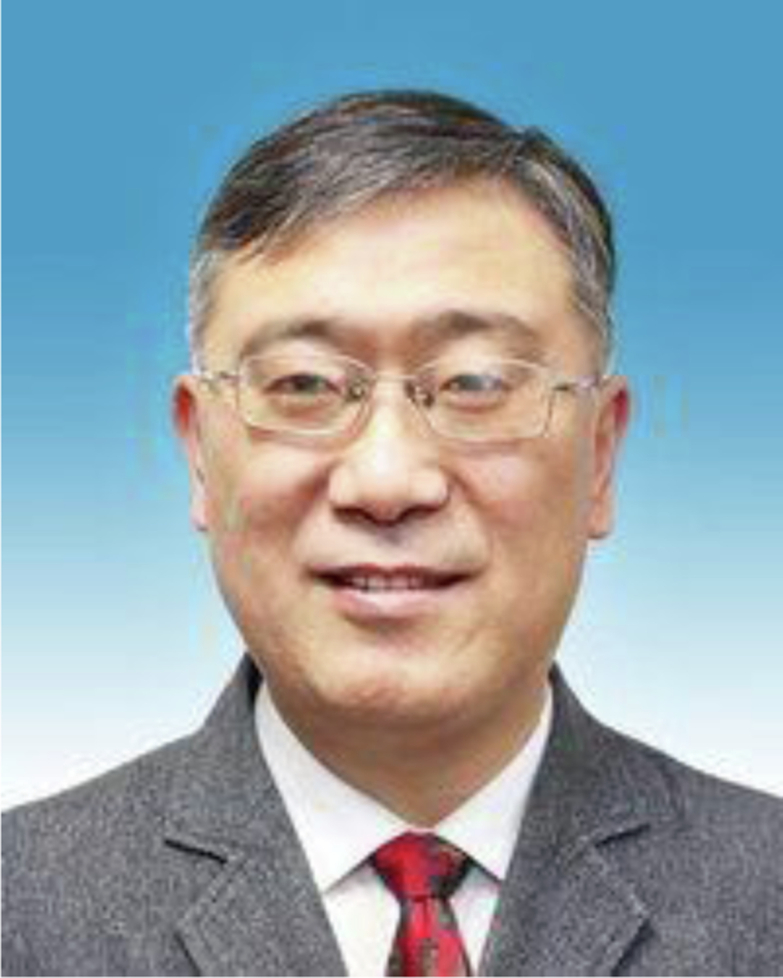



**Short Bio of Prof.** Lei Zhou: Lei Zhou received his Ph.D. in 1997 at Fudan University, and worked as a postdoctoral researcher at Tohoku University (1997–2000) and Hong Kong University of Science and Technology (2000-2004). He joined the Physics Department of Fudan University in 2004, served as Chair of the Physics Department from 2020 to 2024, and became the Vice President of Fudan University in 2024. He is a Fellow of OPTICA, APS, and COS. He works in the fields of Metamaterials and Nanophotonics, has published 4 books, over 230 peer-reviewed papers in scientific journals such as Nat. Mater., Nat. Nano., Light: Sci. & Appl., PRL, PRX, etc., and got 18 invention patents granted. His total citations exceed 23000, and his H-index is 66 (Google Scholar). He has been continuously identified as a “Clarivate” Highly Cited Researcher since 2019. He is the winner of many prestigious prizes, including the second Prize of the National Natural Science Award of China and the first Prize of the Natural Science Award of Shanghai. He is the co-founding-Editor of *Photonics Insights*, and a managing editor of *Nanophotonics* (2019-2025).

**Q1: You have been pioneering at the field of metamaterials and metasurfaces. What first drew you to these areas?**

**A1:** I went through a huge transition in my research focus. During my PhD and early postdoctoral years, I had been working on the theory of electron correlation and magnetism—not even close to electromagnetic waves. But when I moved to HKUST in 2000 for my second postdoctoral period with Prof. Ping Sheng and Prof. CT Chan, things have changed accidentally. I remember one day, Prof. Weijia Wen - our experimental collaborator - fabricated a fractal structure composed of densely packed metallic elements. What fascinated and puzzled us was, light could either fully transmit through or reflect off the structure. Of course, soon after that, history has known Prof. John Pendry’s negative refraction, and Prof. David Smith’s experimental verification. But back then, it took us some time to realize that this was caused by subwavelength resonances. The work got published in PRL in 2002^1^, and we didn’t refer to such structures as ‘metamaterials’, but it eventually became clear that fractal structures are excellent candidates for inducing subwavelength resonances and constructing metamaterials.

So the discovery about fractal structures might be a coincidence, but given that CT’s group had been working extensively on photonic crystals (which are periodic), the emergence of such artificial micro-/nano- structures that could transcend periodicity was truly exciting for all of us. Driven by that passion, my colleague Jensen Li (now a professor at University of Exeter) and I tried to stack positive-ñ and negative-ñ materials, hoping to uncover unusual transmission properties, but I believe you all know from our later publication^2^, it leads to the unexpected zero-ñ gap, which further shifted our way of thinking about photonic crystals, and further led to my research transition towards metamaterials.

**Q2: What’s the most unexpected development in the field of metamaterials?**

**A2:** Honestly, at the very beginning, I was simply intrigued by our unexpected findings, and my expectations on such a classical physics topic were quite limited, as my background was primarily in quantum physics. But as time went on and as I dive into the field, I started to realize how huge potential it holds. Among all the excitement in metamaterials, metasurfaces stand out as the most surprising to me. Though our work^3^ along with Prof. Federico Capasso’s work^4^ has pioneered this field, I personally did not see it coming when I first entered the metamaterials era. The interfacial properties of metasurfaces have introduced a paradigm shift for transcending bulk materials. In particular, when people try to modulate and manipulate light, quite often we expect to make an analogy to existing knowledge in condense matter physics. Yet, metasurfaces have proven to be fundamentally different. That’s why I consider them the most unexpected and groundbreaking development in the field.

**Q3: Thanks to the contributions of many leading scientists like you, metasurfaces have already approached real-world applications? What do you think are the killing application of metasurfaces?**

**A3:** Personally, I will name three applications. First, for military use, scientists have already demonstrated the use of frequency-selective surfaces in the microwave regime. But the uniquely subwavelength properties and active control of electromagnetic waves offered by metasurfaces hold great promise for the microwave range military use, such applications could be very instant. Second, for consumer electronics, the current lenses are still too bulky, especially for our mobile phones, so planar metalens operating in the optical regime could be attractive, but it might take some time. Third, in on-chip photonics - whether for optical computing or information processing- metasurfaces will play a pivotal role in modulating and manipulating light within a compact planar footprint. Their compactness, efficiency, and multifunctionality are valuable for coupling spatial light into or out of photonic chips while manipulating it. Hopefully our work^3^ represents a promising starting point in this direction, and our recent work^5^ provides a possible solution. With growing understanding of metasurfaces and advances in micro- and nanofabrication techniques, I believe on-chip photonics will become one of the major areas for applying metasurfaces.

**Q4: With the platform of metasurfaces, your group has demonstrated several fabulous works of optical controlling and manipulation, including controlling angular dispersions** ^6^ **, circular polarized terahertz waves** ^7^ **, and the generation of complex vectorial optical fields** ^8^ **, what’s your perspective towards metasurfaces and new merging platforms for optical controlling and manipulating, e.g. two-dimensional materials?**

**A4:** I believe they are fundamentally different. Two-dimensional materials offer an exciting new class of material systems, while metasurfaces provide a new paradigm to further design those materials. As I mentioned earlier, when people try to modulate and manipulate light, quite often we expect to make an analogy to existing knowledge in condense matter physics. But metasurfaces match no such analogy and offer an underlying paradigm shift to go beyond the limitations of global sequence, periodicity, and symmetry. This new paradigm is open to and can be integrated with any materials platforms, to unlock entirely new functionalities and offer new freedom of modulation.

**Q5: Do you have any research that was initially overlooked until years later?**

**A5:** Indeed, yes. Back in 2007, we published a work^9^ about using ultrathin anisotropic materials to manipulate polarizations. To the best of my knowledge, it was the first work to exploit the anisotropic interfacial phases to control electromagnetic waves - an idea that later became a foundational principle of metasurfaces. However, in the first two to three years, the work received little attention. It wasn’t until the emergence of [3] and [4] that the broader community began to fully recognize the significance of this concept.

**Q6: In 2020, you derived a theory from first principles for fast designing functional devices, published in** ***Light: Science & Applications***^10^**. And now with the booming needs of complex photonic devices, how do you see this theory can help with the design and reduce computing power?**

**A6:** I believe this is the right time for this work to make a broader impact. Traditionally, the design paradigm for metamaterials - particularly inhomogeneous metasurfaces - has relied heavily on previous experience and extensive parameter scanning. This process is extremely time-consuming and even impractical as device dimensions scale up to the centimeter level or beyond. But with our theoretical framework derived from first principles, one can significantly reduce the parameter scanning range and computational cost when designing. Especially with the rapid advancement of efficient AI tools, this paves the way for a transformative shift in designing complex photonic devices. Admittedly, the derived theory may seem less easy to handle than conventional tools such as FDTD. However, we are actively working to make this framework more comprehensible and accessible to the community.

**Q7: As a leading scientist in both optical physics and optical devices, when you conceive innovative research, are you more driven by curiosity or applications?**

**A7:** I think, at the beginning, it’s always curiosity that drives the work - especially as a physicist. I’ve been constantly trying to uncover the underlying mechanisms and logics behind the unknown. But over time, I’ve become increasingly aware of the gap between scientific capability and real-world applications. As a result, my research has gradually come to be driven by both curiosity and applications, depending on different phases of the research. Take the development of metamaterials as an example. In the early days, we joined the community to construct different types of metamaterials, simply to better understand their properties and capabilities. Then we discovered the gradient index metasurfaces, and we tried to exploit new degrees of freedom - such as polarization control, wave-front control, and time-domain modulation, etc. By then, we were largely curiosity-driven, trying to equip ourselves with new capabilities and tools. Following that, we started to consider potential applications and spent enormous efforts in generating and manipulating vectorial optical fields with tailored needs. After all, electromagnetic waves are inherently vectorial, and their wavefront and polarization distributions can be inhomogeneous. So we proposed a generic approach to design metasurfaces with inhomogeneous full-matrix Jones matrix distributions that can efficiently generate arbitrary vectorial optical fields^8^. Following that, we further brought in cascaded metasurfaces to unlock more freedom in the time domain^11^. At this stage, the motivation became more balanced, driven equally by scientific curiosity and the pursuit of practical utility, and we’d been trying to integrate our different capabilities and tools to cater to the practical needs. As a physicist, I find such application-driven research as challenging as purely fundamental research, and personally I would feel very satisfied to see what I started will beautifully end.

**Q8: You mentioned about the sharp transition of your research focus, how do you look at going into different fields?**

**A8:** I didn’t consciously intend to do that, but as we discussed earlier, it was a natural consequence driven by curiosity. However, when looking back, I feel lucky that I got the opportunity to explore different areas before getting a faculty position, which enriches my background and perspective. And I would also strongly suggest young researchers - who aspire to build a research career - to work on two to three distinctly different topics during their doctoral and postdoctoral years. A multi-disciplinary background often fosters innovation. It’s like the character Jing Guo in the book *The Legend of the Condor Heroes*—he learns from many different masters, absorbs diverse martial arts techniques, and ultimately achieves a comprehensive understanding to become a great master himself. Many well-established scientists have shared the same viewpoints and benefited from this.

For instance, Prof. Shanhui Fan - a renowned scientist in optics and an old friend of mine - proposed the brilliant concept of subambient passive daytime radiative cooling. Such an idea would not have been possible without a solid background in thermodynamics. Personally, I’ve also benefited greatly from multidiscipline. We have talked about our work^10^ earlier, without a background in condensed matter theory, I would never have thought about the tight-binding model or developing its photonic counterpart. Similarly, without a foundation in optics, the idea of leveraging optical scattering would not have occurred to me. In short, this work^10^ could not have happened without a multidisciplinary background. To summarize, I’d like to quote the Chinese idiom “厚积薄发” (hou ji bo fa), which means: one must accumulate knowledge and experience deeply before achieving a major breakthrough. Of course, venturing into new topics can be risky and struggling. For me, I published nothing in the first two years of transition. But it’s better to take those risks in your early career, when the cost of trial and error is lower.

**Q9: As Vice President of Fudan University, you must have been shouldering tremendous administrative responsibilities, but meanwhile you’ve been very active in science, how do you balance your administration and your research?**

**A9:** Balancing both is certainly challenging, but I try to make the most of marginal time and online tools. On one hand, I schedule our weekly group meetings on weekends, as those times are usually quieter and less occupied for me, allowing me to devote more focused energy to research. And I’m truly grateful to my team for generously sacrificing some of their weekend time for me. On the other hand, I try to minimize face-to-face meetings; instead, by using online platforms, I can hold discussions more flexibly and efficiently during otherwise fragmented time slots. Fortunately, I genuinely love science—doing research feels more like relaxation than work. So today you can still see many of the works and projects that I’m directly and heavily involved, which look typically mine. In addition, I maintain an optimistic mindset of seeing administrative responsibilities and research as complementary, just like water and salt, they dissolve into one another. In fact, administrative work has practically helped me better manage and support my research group.

**Q10: How does your administrative mindset evolve with your changed roles?**

**A10:** It’s essential to develop an evolving administrative mindset. When you’re leading a single research group, your primary responsibility is to guide the team forward. However, when you’re entrusted with overseeing many groups, your role shifts—you need to know how to support each group leader effectively. In my view, this means providing a strong and reliable backup to ease their concerns, offering guidance to help them stay on the right path, and granting them enough freedom to explore and forge their own way forward. It also requires knowing what to insist on and what to let go, so that each individual can maximize their strengths and contribute to a collective success.

**Q11: As one of the most prestigious universities in China, Fudan University has recently announced to cut down liberal arts and social sciences enrollment, and in contrast, raising the emphasis of science, engineering, medicine, and interdisciplinary studies. What’s your perspective about this, especially at the turnaround time of Fudan’s 120**^**th**^ **Anniversary?**

**A11:** I think it’s a great move - not only for science, engineering, medicine, and interdisciplinary studies, but also for the liberal arts and social sciences. First, as our President Li Jin has stated in the media and you rightly pointed out, Fudan will strengthen four directions (science, engineering, medicine, and interdisciplinary studies). Together with the liberal arts and social sciences, each of these areas is expected to account for 20% of Fudan’s research portfolio. Second, this initiative will also help elevate Fudan’s already strong liberal arts and social sciences programs to an even higher level of excellence. In particular, with the rise of artificial intelligence, traditional liberal arts and social sciences must undergo transformation—rethinking the traditional notions of knowledge transfer and the way of doing liberal arts and social sciences research. Integrating science and technology into these fields could be highly promising. For example, scientific tools applied in archeology are already proving to be effective. So, this move is not about cutting down the liberal arts; on the contrary, it serves as an incentive to strengthen and modernize them for the new era.

**Q12: Fudan University has recently launched the new College of Future Information Technology** **, which you will lead, what does this school target at, and in your eyes, what are the most promising future information technologies related to optics?**

**A12:** The new College of Future Information Technology reflects Fudan’s ambitious move for new engineering sciences. Before launching it, Fudan has brought in many leading groups and run them rather independently over the past decades. Such efforts by generations of Fudan’s colleagues - reinforced by national and society needs - have finally given birth to this new school. This school will encompass the talents from engineering sciences, application-driven fundamental sciences and multidiscipline, targeting at future industries - information science and technology, especially in aerospace. With my experience in both theoretical physics and application-driven optics, I’m humbled for the opportunity to lead the new school. Hopefully, with our joint efforts, we can pave the way of our fundamental breakthrough towards game-changing technologies in the industry, especially in terms of optical engineering, electrical information and aerospace technologies. We warmly welcome young talents from all over the world to join our Innovation School.

**Q13: What qualities do you look for in a student joining your group?**

**A13:** Firstly, I would name the genuine interest in science as the most important quality. On the research path, one inevitably encounters numerous difficulties, and it is this passion for science - not just desire for success - that sustains you through the challenges. Secondly, I value students who show strong resilience and are able to cope with frustration and stress. Such capability is essential not only for research but also for life. Completing a major project often involves tackling one demanding task after another. You always feel discouraged midway, so it’s important to know how to manage those negative emotions constructively. Thirdly, I appreciate individuals who are motivated to set ambitious goals and organize their schedules effectively. If a student approaches me with a clear vision of what and how they want to do, I would be highly impressed and supportive. Such quality is also a valuable asset in any profession, in my opinion. Of course, no student is perfect—nor was I when I was a student. So rather than expecting students to possess all these qualities from the start, I focus on helping them grow in these areas. For example, during our weekly meetings, each student is required to give a one-minute presentation summarizing what they accomplished in the past week and what they plan to do in the coming week. This practice facilitates their goal-setting and scheduling capability.

**Q14: What advice would you give to early-career researchers facing a lot of pressures in funding, publishing and competition in today’s climate?**

**A14:** I would encourage early-career researchers to focus less on external pressures and competitors, as these are factors beyond your control. Worrying too much about what you cannot influence only leads to constant anxiety, fear of uncertainty, and an unhealthy sense of competition. Instead, turn your attention inward and concentrate on your own growth. If you focus on making steady progress, knowing that you are becoming a better researcher each day, then you regain a sense of control—and with that comes a deeper inner peace. Also as my friend and colleague Prof. Din-Ping Tsai jokingly puts it: ‘Happiness equals gain divided by expectation.’ If you can’t increase your gain, then lower your expectation. I fully agree with this, and raising happiness will for sure benefit your career.
